# Nanoscale visualization of extracellular DNA on cell surfaces

**DOI:** 10.1002/ansa.202000095

**Published:** 2020-09-03

**Authors:** Anita Olsen, Christopher J Ehrhardt, Vamsi K Yadavalli

**Affiliations:** ^1^ Department of Chemical and Life Science Engineering Virginia Commonwealth University Richmond Virginia USA; ^2^ Department of Forensic Science Virginia Commonwealth University Richmond Virginia USA

**Keywords:** atomic force microscopy, buccal cell, extracellular DNA, forensics, palm cell

## Abstract

Nanoscale analysis of extracellular DNA (eDNA) that is present on the surface of cells in trace biological samples can provide insight into the understanding of DNA transfer through touch, and thereby, the role of eDNA is a biologically and forensically relevant phenomenon. While various bulk scale tools and DNA analysis can be used to quantitatively obtain this information, obtaining a three dimensional (3D) visualization of the eDNA can provide a unique look into the spatial and temporal dynamics at the cellular level. In this study, we show how atomic force microscopy (AFM) can be integrated with optical microscopy to visualize the distribution of surface associate eDNA at a single cell level. Using a nucleic acid fluorophore such as Diamond™ Dye, the surface eDNA can be observed and quantified using fluorescence microscopy. This informational channel can then be overlaid with surface topography and cellular elasticity to provide structural visualization. Finally, chemical force spectroscopy can be used to obtain the distribution of surface‐associated eDNA on the cell surface at the molecular level. Such integrated techniques can enhance understanding of the biological role of eDNA, and can also be potentially valuable for investigating challenging trace samples, containing very few cells for various analyses.

## 1 INTRODUCTION

The collection and analysis of epithelial cells has emerged as one of the cornerstones of forensic analysis. Humans shed ∼400 000 cells daily. Thus, the act of picking up an object or touching a surface (either inanimate or living) can provide a source of useful evidence. Epithelial, touch, saliva, and contact evidence contain variable amounts of DNA resulting from the transfer of cells or extracellular DNA to an object surface.^1^, ^2^ This has the potential to be useful evidence, for instance in cases of violent crime involving a close physical interaction between two individuals. Understanding the mechanisms of DNA transfer through touch and contact, while developing methods to maximize the level of DNA recovery from contact surfaces is therefore a continuing priority.^3^

The DNA found in a contact sample was thought to be primarily based on the number of cells shed naturally from the outermost layer of skin or mouth. Recent studies have shown that such samples can also contain “cell‐free” or extracellular nucleic acids (sometimes referred as CNA, cfDNA, or eDNA), in contrast to intracellular DNA.^4^, ^5^ For many samples, this extracellular DNA constitutes an overwhelming majority of the total recoverable DNA and, importantly, may not yield full STR profiles.^6^ The precise mechanism by which the “touch DNA” occurs, as well as the role of extracellular DNA in contributing to the quality of a DNA profile generated from samples continues to be a subject of debate and analysis.^7^ Of particular interest is the extracellular DNA (herein referred to as eDNA) that is attached to the cell surface, as opposed to free, unassociated DNA. Nanoscale analysis of this eDNA in trace biological samples (specifically touch epithelial cells) can provide insight into the understanding of DNA transfer through touch, and thereby, the role of eDNA is a biologically and forensically relevant phenomenon.

The atomic force microscope (AFM) has rapidly emerged as an important, widely used tool in cellular nanoscale analyses.^8^, ^9^, ^10^ While primarily developed as an imaging tool,^11^ enhancements have permitted the detection and functional analysis of cell surface receptors, and quantitative measurements of mechanical properties using force spectroscopy.^12^, ^13^ The AFM has only recently been finding its way for forensic applications primarily in the examination of evidence such as blood stains, forged documents, hair samples, ammunitions, and explosives.^14^, ^15^ In an earlier report, we showed how cell surface‐associated eDNA, as well as the morphology, and mechanical properties of individual epithelial cells can be visualized using AFM as an imaging and nanomechanical tool. 3D nanoscale topography, cellular elasticity, and chemical force spectroscopy were used to visualize the distribution of surface‐associated eDNA on the cell surface.^16^ In these studies, chemical force microscopy was conducted with an AFM with a lactoferrin functionalized cantilever to identify individual eDNA on the cell surface. However, in order to be more accessible to the researcher, it is important to visualize the eDNA at the level of the entire cell, while being able to combine data streams with other, more widely available microscopic techniques.

In this study, we demonstrate the integration of optical microscopy with AFM for the visualization of topographical distribution of eDNA using fluorescence imaging. While various studies have shown the utility of combined AFM and fluorescence imaging, they primarily focused on intracellular targets. Here, we show how a surface‐attached target can be interrogated using this strategy, specifically providing a direct visualization of the surface‐attached eDNA. We utilize nucleic acid dyes that have been shown to be viable indicators of cellular material in palm and lip prints.^17^, ^18^ Epithelial cells obtained from touch and buccal samples were labeled with the DNA specific fluorescence probe and the resulting images were overlaid on topographical AFM images to show 3D distributions and concentrations of DNA on or within the cell. The fluorescence intensities are then compared between cells with and without DNase treatment that removes eDNA from the surface of the cell.

These studies can permit various micro‐ and nanoscale measurements to identify and elucidate attachment mechanisms of eDNA. They can also assist in bridging the gap between single cell analysis conducted with AFM and bulk studies such as qPCR and flow cytometry. Optical fluorescence microscopy and flow cytometry provide two‐dimensional shape and fluorescence information that can be directly correlated. DNA quantification from sample washes and cell pellets can be combined with cell concentration data to determine the distribution of intra‐ and extracellular DNA within samples. The results can then be compared across donors with AFM/optical microscopy of individual cells to observe variance and behavior of eDNA within touch samples. Interestingly, it has also been shown that eDNA plays a pivotal role in the establishment, maintenance, and perpetuation of bacterial biofilms.^19^ Discoveries made with this investigative technique will not only enhance understanding of the biological role of eDNA, but can also be potentially valuable for investigating challenging trace samples, containing very few cells for various analyses.

**Lay description**

Forensic evidence from touch or saliva typically contains a small number of cells from the donor. Recently, it has been found that these cells have extracellular DNA that is anchored to the surface of the cells. This eDNA is distinct from the DNA that is contained within the cells. Understanding and visualizing the spatial and temporal profiles of this eDNA can be useful for both fundamental as well as applied science (eg, forensic attribution). Here, we show a new way of combining fluorescence imaging with atomic force microscopy of single cells that shows the presence and spatial location of eDNA on the cell surface. This provides a unique visualization of the cell surface and how the eDNA is distributed and can potentially change depending on cell type (eg, buccal cells or palm cells) or with time/external environmental conditions.

## 2 MATERIALS AND METHODS

### 2.1 Materials

Diamond™ Nucleic Acid Dye (DD) was purchased from Promega (Madison, WI). Poly‐l‐lysine hydrobromide and DNase II enzyme were purchased from Sigma‐Aldrich (St. Louis, MO). Phosphate‐buffered saline (PBS, pH 7.4; 11.9 mM phosphates, 137 mM sodium chloride, and 2.7 mM potassium chloride) and ethanol (200‐proof) were purchased from Fisher Scientific (Waltham, MA). Epithelial cell samples were collected from volunteers using an Institutional Review Board (IRB) approved protocol (HM20000454_CR5). Buccal cells were collected from individuals by rubbing the insides of both cheeks with a dry sterile cotton swab for 5 s and then eluted into 1 mL TE buffer. Touch epidermal cells were obtained by having individuals grip a sterile polypropylene conical tube for ∼5 min. They were collected from the surface using a wet followed by dry cotton swab and eluted in 1 mL TE buffer. Any residual solution still in the swab was collected by centrifuging the swab tip in a spin basket and added to the sample. Both buccal and touch epidermal cells were washed once to remove any supernatant containing eDNA, leaving only attached eDNA on the cell surface. This was accomplished by centrifuging at 10 000 × *g* for 2 min to pellet cells, removing the supernatant by pipetting, and resuspending cells. One milliliter TE buffer was used for buccal cells and 100 µL for palm cells.

### 2.2 Cell collection and dye staining

A range of nucleic acid dyes with known mechanisms were compiled to investigate alternatives for analyzing palm cells (Table S1).^20^, ^21^ From this group, Diamond™ Nucleic Acid Dye (DD), GelRed™ (GR), GelGreen™ (GG), SYBR Green I (SG), and 2 dimeric cyanine nucleic acid stains (TOTO‐1 and YOYO‐3) were initially chosen to find the proper balance between range of fluorescence, eDNA‐specific labeling, cost, and ease of use.^22^, ^23^ The sensitivity of GR, GG, and SG and DD are roughly similar (tested down to 0.5 ng levels with the possibility of even lower limits of detection).^24^ SG was chosen as a comparable cell‐permeable dye for DD and cell‐impermeable intercalating dyes, GR, GG, TOTO‐1, and YOYO‐1, were chosen to reduce fluorescence interference from intracellular DNA. GR and GG are nontoxic, inexpensive, and stable at room temperature.

Epithelial cells were collected by pressing a finger or thumb onto a glass surface for 10 s. The following protocols were used: a 1/100 dilution of SG in 1× PBS was applied to directly applied to a fingerprint, incubated for 30 min in the dark, then rinsed with 2 mL PBS. For GR and GG, 200 µL of a 3× dilution in sterile H_2_O was applied, incubated at 40 °C for 5 or 30 min to test permeability, and rinsed with 2 mL of sterile H_2_O. TOTO‐1 and YOYO‐3 had concentrations of 2.4 nM in PBS and 200 µL were applied to fingerprint slide, incubated for 20 min, and rinsed with 2 mL PBS.

### 2.3 Fluorescence imaging

Fluorescence images were taken across print samples varying light intensities for 10–30 cells per sample. Since palm cells emit some autofluorescence, images of untreated cells were taken as a control. The mean fluorescence intensities of each sample and intensity were obtained using cell analysis software for comparison. CellProfiler software was used to detect and measure the mean intensity of fluorescence in individual cells.^25^, ^26^ The cells were identified and masked by fluorescence intensities above background, and contamination was filtered out by size. Light intensities were varied due to a dramatic difference between DD, GG, and GR intensities and SG, TOTO‐1, and YOYO‐1 that required much higher intensity light excitation for detectable fluorescence. Measurements of fluorescence intensity is averaged across cell populations of specific dyes and the intensities are compared to determine a proper intensity range for use in DNase II treatment experiments.

### 2.4 DNase II comparison

Buccal cells were collected as previously described and were divided into two aliquots, providing an untreated sample and one treated with DNase II. All samples, with or without DNase treatment, were then stained with DD by adding 100 µL of dye to solutions, vortexing for 10 s, and incubating in room temperature for 30 min. Cells are pelleted, supernatant removed, and resuspended in 1 mL FACS buffer. Touch cell collection protocol was modified to increase cell adherence for AFM scanning by applying a direct thumbprint to glass slides, modified with 0.05 mg/mL poly‐l‐lysine hydrobromide, with moderate pressure for 10 s. Two prints are taken simultaneously and one is subjected to an enzymatic reaction with DNase II (100 µg/mL in 1× PBS). The treated print is soaked in 500 µL of DNase II solution for 1 h and all glass slides, with treated and untreated prints, are rinsed 3× with 1 mL sterile H_2_O. DD solution was applied and left to soak for 30 min in the dark at room temperature and rinsed once with 1 mL sterile H_2_O. Optical and fluorescence images were taken and compared using cell analysis software to determine intensity variance between untreated and DNase II treated cells.

### 2.5 AFM/optical microscopy overlay

Glass slides were modified using a poly‐l‐lysine fixation method to immobilize cells during AFM imaging.^27^ Glass was cleaned with ethanol and immersed for 10 min in a solution of 0.05 mg/mL poly‐l‐lysine hydrobromide and 10 mM Tris (pH 8.0). The slides were covered, dried vertically overnight at room temperature, and used within a week. Approximately 100 µL of the dyed solutions were deposited onto modified glass slides, incubated for 30 min, and rinsed with 3 mL sterile H_2_O. The microscope objective was calibrated using AFM software for precise image overlay on topographical AFM scans. Both optical and fluorescence images were taken in the same location and are overlaid using image analysis software. Non‐contact mode topographical scans of the same cells were taken with an AFM (MFP‐3D Bio, Asylum Research) using AC240TS cantilevers (Olympus), cleaned with high‐intensity UV light to remove organic contamination. The final overlay of the optical/fluorescence image and topographical AFM images was performed in IgorPro software and 3D images of fluorescence distribution could be created.

## 3 RESULTS AND DISCUSSION

It is frequently assumed that “touch DNA” is derived from shed nucleated cells and stripped nuclei dispersed among mostly anucleate corneocytes, typically found on the surfaces of the outermost layer of the epidermis.^28^ The presence of eDNA is often considered to be the result of cell apoptosis remnants that are secreted in bodily fluid. Indeed, work from our group has shown that a considerable portion (>85%) of DNA from a touch sample is eDNA.^7^ Proportions of extracellular and intracellular DNA have varied widely across recent studies.^4^, ^29^ In another recent study, we have reported on the total abundance and proportion of eDNA across donors as well as the reproducibility of eDNA yields across multiple sampling events from the same donor. An outstanding question concerns the “differentiation” between cellular and extracellular DNA, and their relative contributions to the total genomic content of a sample. How much of this DNA is physically associated with the surface of the cell (cell surface bound) versus “free” or circulating, and how is the surface‐bound DNA attached to the epithelial cell? This is important because the manner in which extracellular DNA is associated to the cell surface can influence strategies to enhance the total recovery of DNA from samples and well as efforts for front end cell separation of DNA mixtures. Several groups have focused on recovering and quantifying the portion of non‐bound eDNA.^4^, ^6^, ^28^ From a forensic perspective, this has implications in the collection of trace samples and the generation of a DNA profile based from contact biological samples. The cell‐surface bound eDNA also plays important roles in a clinical context and in biofilms.^30^, ^31^ However, to date, there have been limited studies on the profile and distribution of the DNA on the surface of cells, and has remained largely unexplored.

**FIGURE 1:**
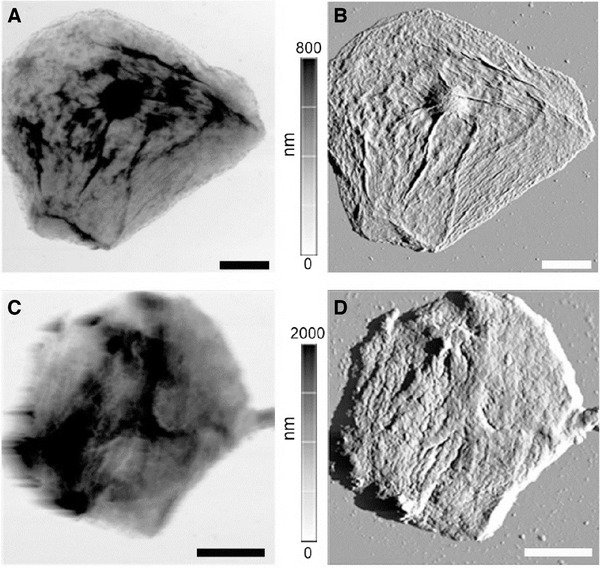
AFM imaging of epithelial cells from buccal cells (A, B) and palm (C, D). The panels on the right (B, D) show the deflection error signal that can show the ultrastructure, whereas the panels on the left (A, C) show the topography of the cells. Scale bars on all panels = 10 µm

Atomic force microscopy (AFM) provides a non‐destructive approach to study both the cell surface and visualize the extracellular, attached eDNA. It is possible to non‐destructively investigate single cells at the nanoscale to obtain a wealth of information, including but not limited to –morphology of the cell, mechanical properties of the cell, and the presence and distribution of specific biochemical targets on the cell surface. Probes attached to AFM cantilevers can be used to locate cognate oligonucleotides on the epithelial cell surface. By combining “force recognition” with high resolution microscopy, it is possible to obtain the spatial resolution of surface‐immobilized DNA on cells.^32^ This technique is sensitive, yet non‐destructive, allowing us to probe each cell repeatedly and over time, providing a look at spatial and temporal dynamics of a collected sample. As seen in Figure 1, epithelial cells from both palm and buccal samples collected from human donors may be observed at the single cell level. Micro and nanoscale features may be obtained with unprecedented resolution. Buccal cells are nucleated in comparison to the cells acquired from the surface of the palm.

In this study, the eDNA is visualized at multiple length scales (nanoscale to the level of single cells at the microscale) using an integrated atomic force microscope and optical fluorescence technique. The AFM used in this study is combined with an optical microscope, to enhance the specificity of these interactions and to correlate single cell analysis with bulk analysis (Figure S1 shows the integrated modality). DNA specific fluorescent probes were applied to epithelial cell samples and mapped. This allows for an overlay of fluorescence with topographical maps on the same cell for a unique visual and informational perspective.

### 3.1 Optimization and comparison of DNA binding dyes

Initially, we focused on finding an optimal DNA binding dye that could be used for labeling the extracellular eDNA. Measurements of fluorescence intensity was averaged across cell populations of specific dyes and the intensities are compared to determine a proper intensity range for use in our imaging experiments. A range of nucleic acid dyes was compiled to investigate various options for analyzing palm cells (Table S1).^20^, ^21^ From this list, Diamond™ Dye (DD), GelRed™ (GR), GelGreen™ (GG), SYBR Green I (SG), and two dimeric cyanine nucleic acid stains (TOTO‐1 and YOYO‐3) were chosen for optimization based on range of fluorescence, eDNA‐specific labeling, cost, and ease of use.^22^, ^23^ Fluorescence images were taken across samples stained with different types of dyes by varying light intensities for 10‐30 cells per sample. Since palm cells emit some autofluorescence, images of untreated cells were taken as a control. Table 1 shows the comparison based on intensity of observed fluorescence. The cells were identified and masked by fluorescence intensities above background, and contamination was filtered out by size. The mean fluorescence intensities of each sample and intensity were obtained using CellProfiler software.^25^, ^26^ One of the difficulties encountered with the palm cell measurements is a very high fluorescence intensity. Thus, either the dye concentration or the light intensity needs to be adjusted for ideal visualization. It was necessary to vary light intensities (scaled from 1 to 4, with 1 being the lowest and 4 the highest intensity of excitation) due to the clearly observable differences between the dyes (Figure S2 shows the effect of the changing intensity on the observed fluorescence). TOTO‐1 and YOYO‐1 required much higher intensity light excitation (4) for detectable fluorescence that was at the level of cell autofluorescence, and were not considered further. Cell staining was visible with similar intensity above background fluorescence for SYBR Green I at high intensity excitation (4) comparable to GelGreen at low intensity excitation (1). Thus, it was not considered optimal either.

**TABLE 1:** Table of nucleic acid dye comparison studied across several cells

Nucleic acid probe	Incubation time	Excitation intensity	Donor ID	Number of cells	Mean ± SD
Diamond Dye	30 min	1	L18	27	0.72 ± 0.12
GelRed	30 min	1	L18	26	0.63 ± 0.08
GelGreen	30 min	1	H01	26	0.29 ± 0.11
SYBR Green I	30 min	4	P24	14	0.31 ± 0.06
TOTO‐1	20 min	4	L18	14	0.19 ± 0.06
YOYO‐3	20 min	4	P24	13	0.16 ± 0.09
**Control**	**N/A**	**4**	**P24**	**14**	**0.20 ± 0.05**

**FIGURE 2:**
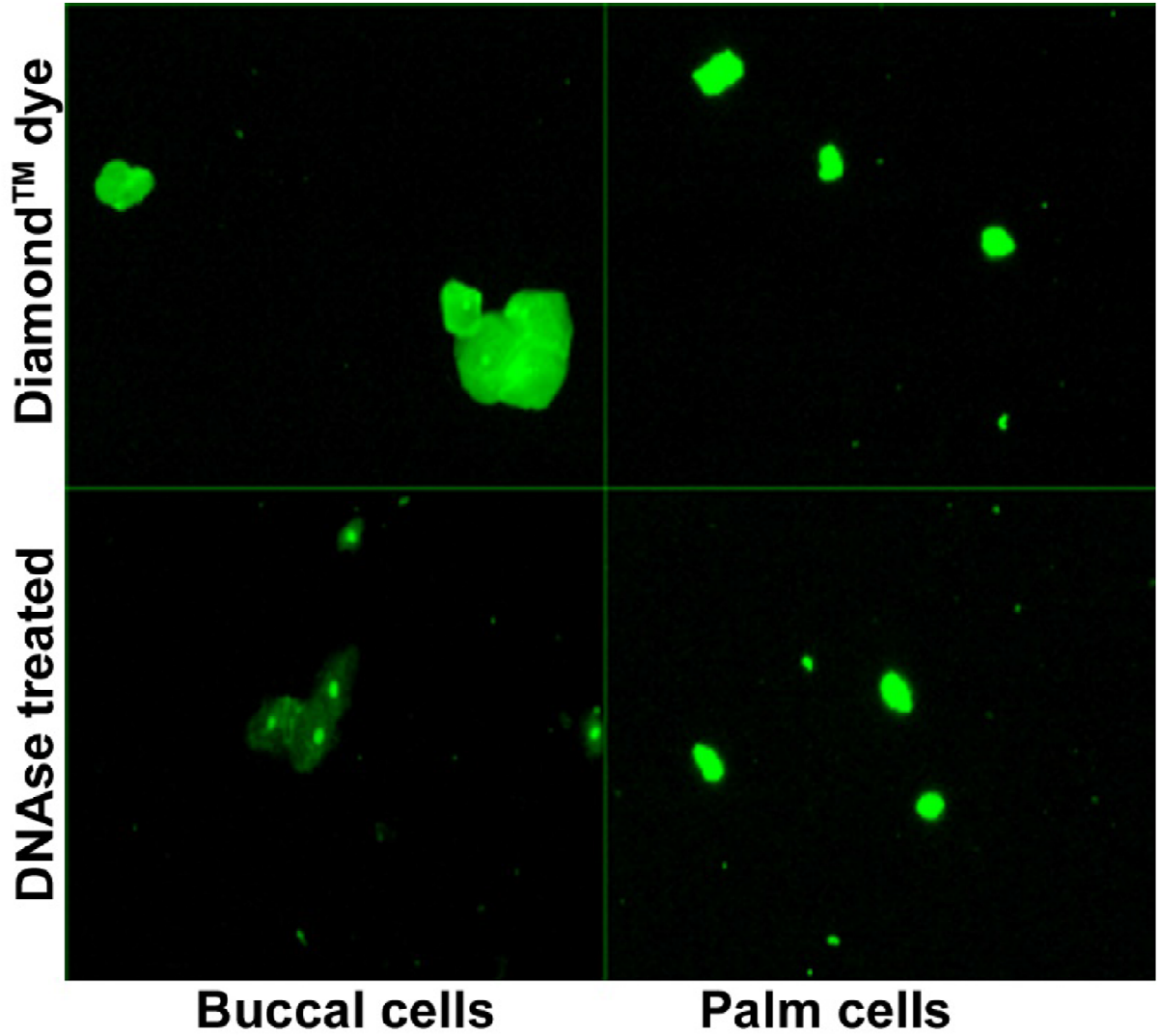
Image of cells stained with Diamond Dye followed by treatment with DNase II. A significant drop in fluorescence is indicative of the removal of surface eDNA

While the staining mechanism of DD is proprietary, and primarily designed to stain nucleic acids in electrophoresis gels, we have observed its suitability for tagging the cellular DNA as well. However, based on its intended use, we were interested to verify if the dye is only staining nucleic acids, or if there is non‐specific staining. Figure 2 shows fluorescence images of buccal and palm cells from single donors (anonymized as D02 and L18, respectively). As expected, the palm cells, being keratinized show a lower level of fluorescence. Following imaging of the cells, the samples were treated with the enzyme DNase II in order to remove any surface eDNA. This data is visually observed and can also be quantified as seen in Table 2. The presence of residual fluorescence shows that the dye does penetrate into the cell, lighting up the intracellular DNA in addition to the eDNA. However, there is a significant drop in fluorescence before and after treatment. This difference is pronounced in the case of buccal cells. In the case of palm cells, a slight increase is noted. However, this is within the error of the measurement, indicating that the fluorescence was essentially unchanged.

**TABLE 2:** Average mean intensity of buccal and palm cells showing untreated cells and cells treated with DNase II, stained with Diamond Dye. In the table below, palm cells stained with GelGreen

Donor ID	Sample	Mean and SD
Buccal cells		
D02	Control	0.78 ± 0.05
	DNase II	0.57 ± 0.13
Palm cells		
L18	Control	0.72 ± 0.12
	DNase II	0.82 ± 0.04
Palm cells		
I66	Control	0.34 ± 0.12
	DNase II	0.34 ± 0.10
G30	Control	0.36 ± 0.15
	DNase II	0.47 ± 0.08
N90	Control	0.38 ± 0.10
	DNase II	0.27 ± 0.17

Both GelGreen and GelRed dyes are also noted to be optimal for observing fluctuations in relative intensity with minimal preparation time and cost. These dyes are also suitable for observations of the cell surface DNA. GelGreen™ was studied as an option in these experiments since it can be used with the same filter set as DD. This is also an excellent choice for use in experiments and to compare between untreated cells and cells treated with DNase enzyme. Only palm cells from three different donors were studied. No significant difference in fluorescence was noted before and after enzyme treatment. For the overlay with the AFM images, DD was used. In summary, both Diamond™ Dye and GelGreen™ represent two good choices that can be used with a standard off‐the‐shelf FITC filter cube (excitation filter 480/30 nm, 505 nm dichroic, and 535/45 nm emission filter) in a fluorescence microscope.

### 3.2 AFM overlay

One question of interest in sample analysis is to differentiate the DNA that is anchored on the cell surface, to the DNA that is inside the cell, and any DNA that may be freely floating in the forensic sample. While the latter two can be obtained by PCR and other biochemical techniques, one way to probe the anchored eDNA is to directly locate it on the surface. Prior research from our group has suggested a way to visualize the physical attachment of eDNA to the surface of buccal and palm cells using lactoferrin as a receptor that can bind to the surface attached eDNA. This is shown in Figure S3. As a non‐destructive probe, the DNA on the cell surface is not dislodged, implying that the cell can be imaged several times.

**FIGURE 3:**
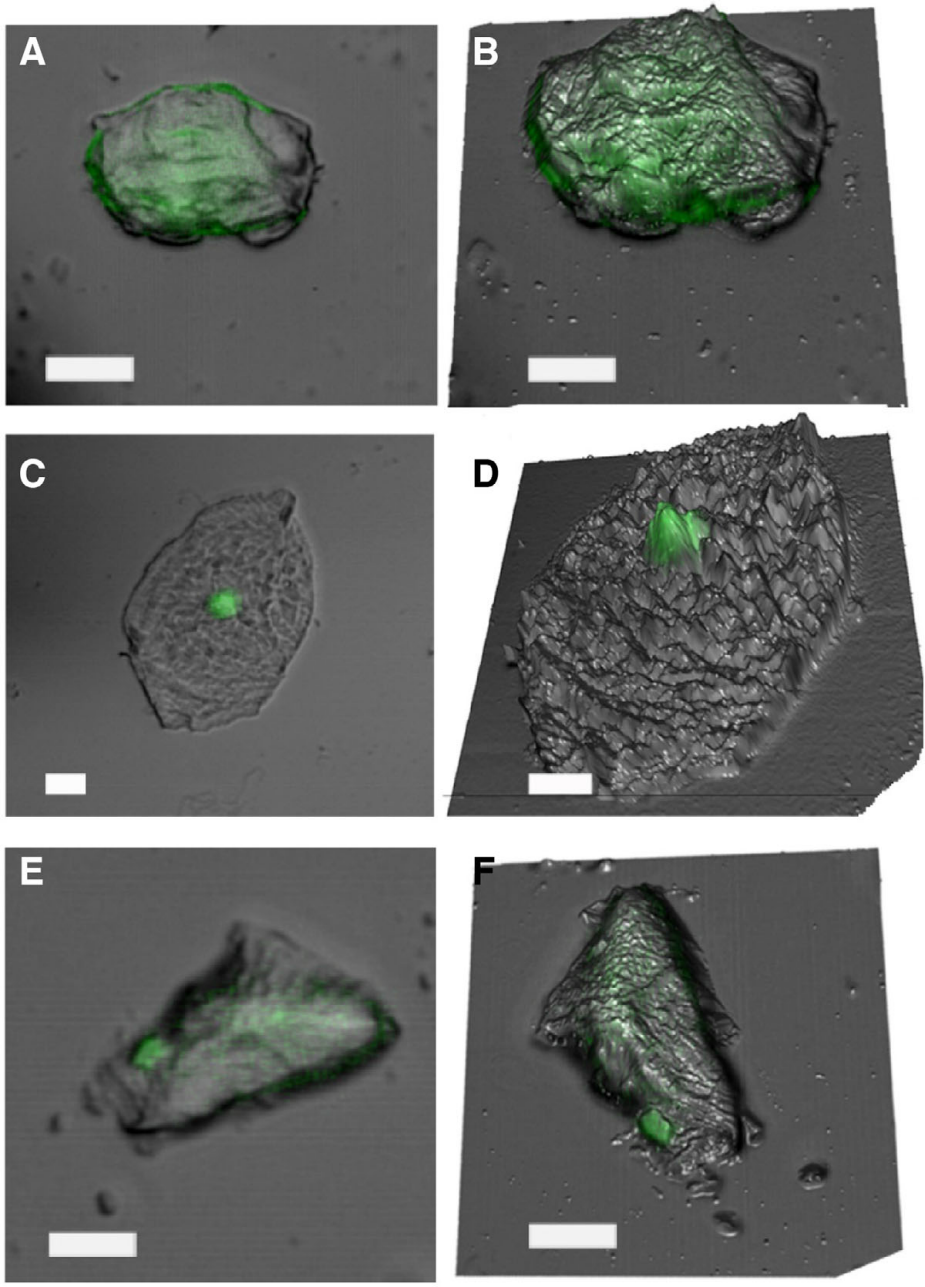
Three dimensional overlay of the fluorescence image with the AFM image showing the spatial location of the fluorescence on epithelial samples. Panel (A) shows palm cells whereas panel (C) shows buccal cell samples. Panel (E) shows cells obtained from a “touch” sample, representative of a palm cell. Panels (A), (C), and (E) are the optical microscope images overlaid on the 3D topography (B), (D), and (F), respectively. Scale bars = 10 µm on all panels

3D images of buccal and palm cells were obtained using an overlay of 2D fluorescence and optical images on topographical images taken with AFM. This is a visual demonstration of the single cell mapping capabilities of this technique. Figure 3 shows the 2D and the 3D visualizations of the integrated topography and fluorescence channels. In each image, the fluorescence image was overlaid on the AFM image taken at the same time. Images of buccal cells show clear morphology of a nucleus that correlate with area of fluorescence indicating presence of DNA. Diffuse or little fluorescence was observed outside the nucleus. In contrast, epidermal cells showed more diffuse fluorescence, characteristic size/morphology with corneocytes. For the first time, these images show a 3D visualization of the presence of eDNA on the cell surface via fluorescence. These can be correlated with force spectroscopic data to indicate the eDNA at the single cell level.

**FIGURE 4:**
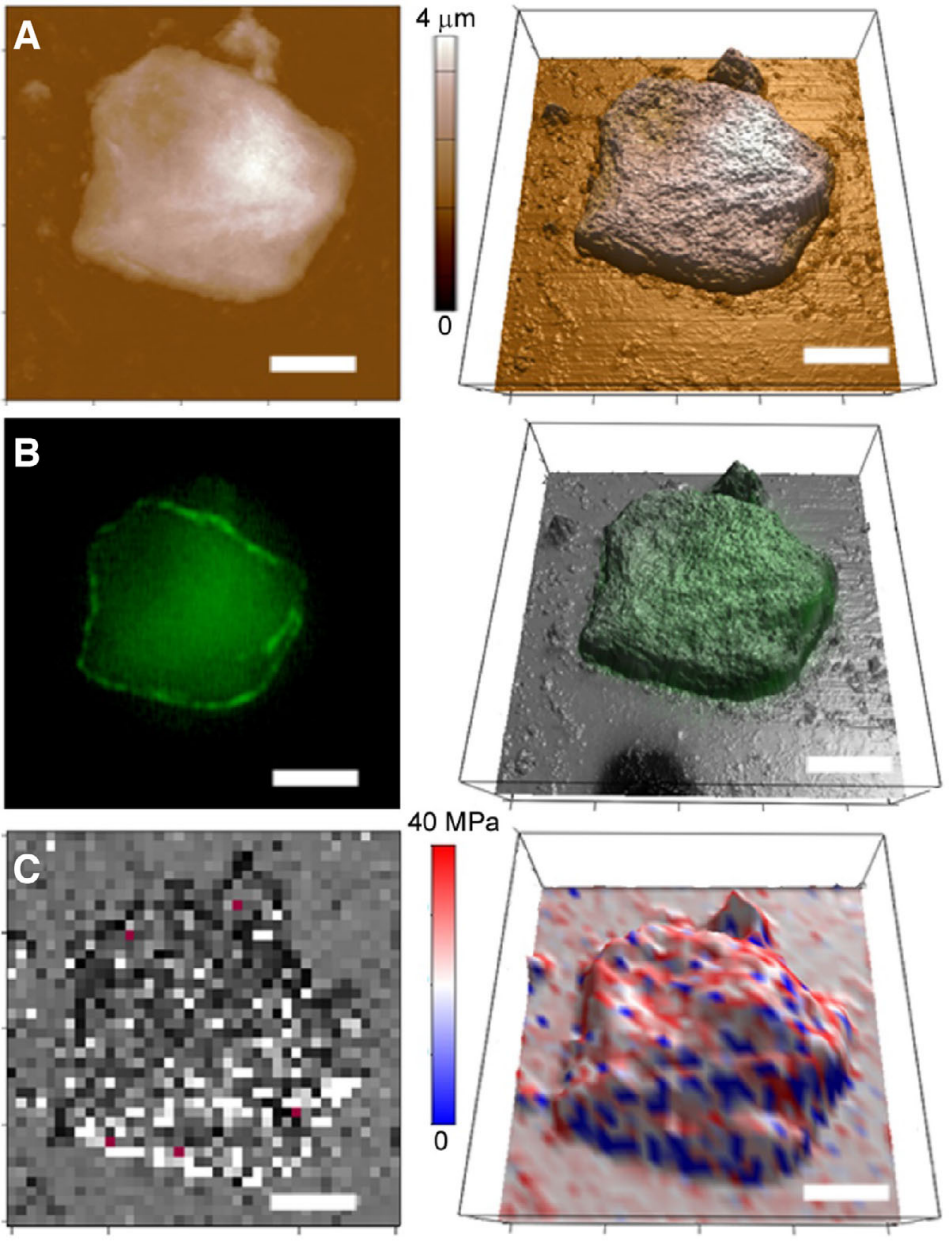
A, Topographical AFM image showing the 2D (left) and 3D visualization (right). B, GelGreen fluorescence image (left) with the overlay on the 3D AFM visualization (right) showing the spatial locations of eDNA. C, AFM elasticity map (left) with the 3D AFM visualization (right) showing the variation of mechanical properties (soft – blue to stiffer – red)

The AFM is a versatile tool that permits us not only to characterize cellular surfaces with nanoscale resolution and 3D imaging as shown above, but also measure their nanomechanical properties.^33^ This can be potentially useful in measuring the changes in stiffness of cells over time or to distinguish between visually similar cells obtained at different locations. Using the AFM tip as an indenter, quantitative information on the elasticity of the sample can be obtained in a non‐destructive fashion at a cellular level.^34^ Figure 4 shows the 3D topography, fluorescence overlay, and the 3D representation of cell elasticity obtained from indentation of the cell sample. Here, the same epithelial cell obtained from a palm sample is shown with the three channels of information. The presence of the surface DNA can be visualized in relation to topographical features on the cell surface. In addition, by combining with nanomechanical and force spectroscopic data, the specificity of the DNA labeling can be verified. This can be further extended to other surface biomarkers, making this a high resolution tool to characterize cells at the single cell level.

## 4 CONCLUSIONS

The development of strategies to identify and visually locate DNA on the surface of cells, as well as observe the difference of the “anchored” eDNA has the potential to provide valuable information in constructing profiles based on collected cellular evidence. These cells may be obtained from the cheek (eg, saliva) or palm epithelial cells (eg, touch), which are potentially important in investigations. By using a combination of atomic force microscopy with fluorescence microscopy, 2D information can be combined with 3D data to obtain a unique visualization of the surface eDNA. Diamond™ Dye and GelGreen™ are useful and easy to use fluorophores to visualize the surface eDNA. Since fluorescence microscopic tools are widely available in comparison to techniques such as AFM, such information can result in wider adoption for high resolution analysis of scarce cellular samples. Characterizing epithelial cells from contact samples could uncover new information and develop ultrasensitive tools for cell attribution genomic characterizations, and precise analysis of eDNA.

## AUTHOR CONTRIBUTIONS

All authors were involved in the design of experiments. Data acquisition was performed by AO. VKY and CJE wrote the manuscript.

## Supporting information

Supporting Information
