# Corrigendum

**DOI:** 10.1002/ansa.202100900

**Published:** 2021-02-17

In Olsen et al.^1^ the permission statement for Figure 1 was omitted from the published manuscript.

The correct permission statement is:

*Figure 1 is reprinted from Forensic Science International, 277, Congzhou Wang, Cristina E. Stanciu, Christopher J. Ehrhardt, Vamsi K. Yadavalli “Nanoscale characterization of forensically relevant epithelial cells and surface associated extracellular DNA”, 252‐258, Copyright (2017), with permission from Elsevier*.

We apologize for these errors.
