# Supplementary material for: Nanoscale visualization of extracellular DNA on cell surfaces
Source: Anal Sci Adv. 2020 Sep 3;1(3):194–202. doi: 10.1002/ansa.202000095 (PMC10989130; doi:10.1002/ansa.202000095)
Supplement: Supplementary file 1 — Supporting Information [file ANSA-1-194-s001.docx]

**Supporting Information for manuscript:**

**Nanoscale visualization of extracellular DNA on cell surfaces**

Anita Olsen,^1^ Christopher J Ehrhardt,^2^ Vamsi K Yadavalli ^1, *^

^1^ - Department of Chemical and Life Science Engineering,

^2^ – Department of Forensic Science

Virginia Commonwealth University, Richmond VA 23284, USA

^*^ - Corresponding author: Phone: 1-804-828-0587

Email **:** [vyadavalli@vcu.edu](mailto:vyadavalli@vcu.edu)

**Table S1 – Some common nucleic acid dyes and their characteristics.**

| **Dye** | **Mechanism** | **Excitation**  **(nm)** | **Emission**  **(nm)** |
| --- | --- | --- | --- |
| Diamond Dye | external groove binding | 494 | 558 |
| Hoechst 33258 | groove binding, AT-selective | 352 | 461 |
| Hoechst 33342 | groove binding | 350 | 461 |
| DAPI | groove binding, AT-selective | 364 | 454 |
| Propidium Iodide | intercalating | 535 | 617 |
| Ethidium bromide | intercalating | 300, 360 | 590 |
| SYBR Green I | intercalating | 494 | 520 |
| PicoGreen | intercalating | 500 | 523 |
| GelGreen | intercalating | 495 | 520 |
| GelRed | intercalating | 300,250 | 600 |
| BOBO-1 iodide | intercalating | 442 | 481 |
| BOBO-3 iodide | intercalating | 568 | 602 |
| POPO-1 iodide | bis-intercalation | 436 | 456 |
| POPO-3 iodide | intercalating | 534 | 570 |
| TOTO-1 iodide | bis-intercalation | 514 | 533 |
| TOTO-3 iodide | intercalating | 633 | 660 |
| YOYO-1 iodide | bis-intercalation | 488 | 509 |
| YOYO-3 iodide | intercalating | 594 | 631 |

**
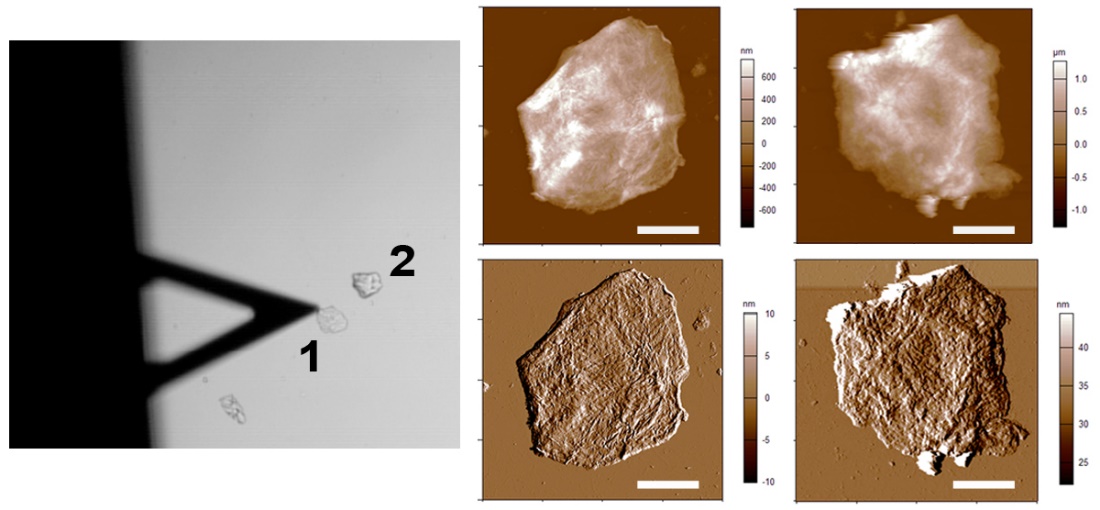
**

**Figure S1 –** Imaging of the cell samples using integrated microscopy and AFM. In this example, two palm cells are imaged. The top and bottom AFM images show the topography and deflection of the cells (1) and (2) respectively. The scale bars = 20 µm on all panels. The inverted fluorescence microscopy channel is below the plane of view.


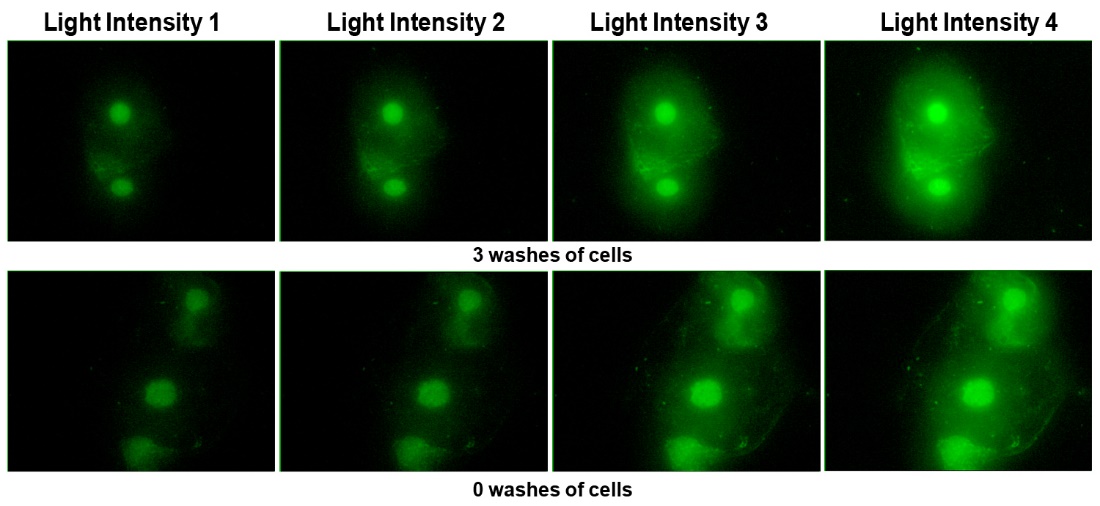


**Figure S2 –** The effect of changing the incident light intensity on the observed fluorescence of cell samples (buccal cells shown in this case with and without washing of water). While increased intensity results in increased signal, it also increases the susceptibility of photobleaching and damage.


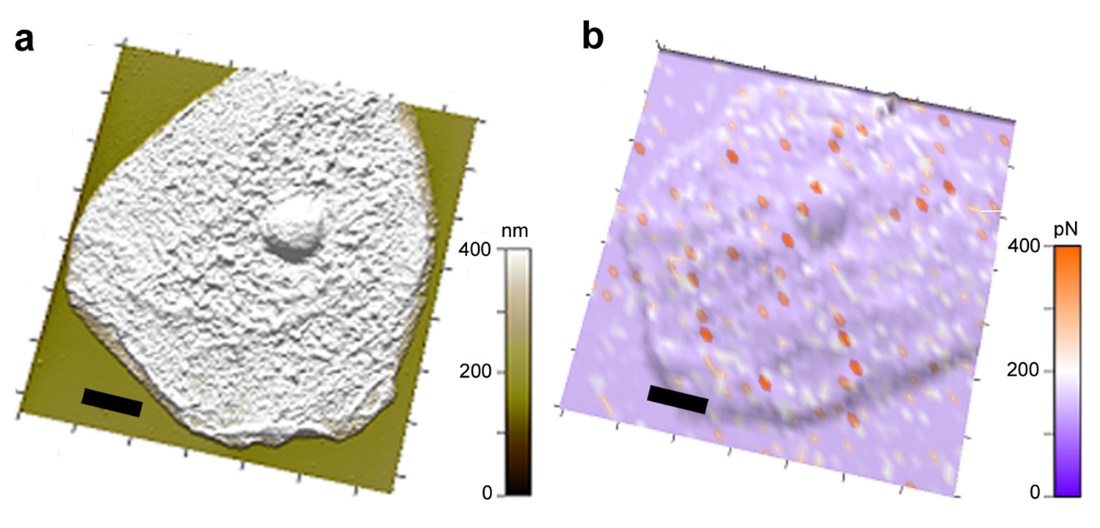


**Figure S3 –** Force spectroscopy showing the position of the eDNA on the cell surface. In this experiment, the cell was probed with a lactoferrin modified AFM tip. The lactoferrin binds to DNA on the cell surface.
